# Biotechnological potential of rhizobial metabolites to enhance the performance of *Bradyrhizobium* spp. and *Azospirillum brasilense* inoculants with soybean and maize

**DOI:** 10.1186/2191-0855-3-21

**Published:** 2013-04-17

**Authors:** Bettina Berquó Marks, Manuel Megías, Marco Antonio Nogueira, Mariangela Hungria

**Affiliations:** 1 Embrapa Soja, C.P. 231, 86001-970, Londrina, Paraná, Brazil; 2 Department of Microbiology, Universidade Estadual de Londrina, Cx. Postal 60001, 86051-990, Londrina, Paraná, Brazil; 3 Universidad de Sevilla, Departamento de Microbiología y Parasitología, Apdo Postal 874, 41080, Sevilla, Spain

**Keywords:** *Azospirillum*, *Bradyrhizobium*, *Glycine max*, LCO, *Rhizobium*, *Zea mays*

## Abstract

Agricultural sustainability may represent the greatest encumbrance to increasing food production. On the other hand, as a component of sustainability, replacement of chemical fertilizers by bio-fertilizers has the potential to lower costs for farmers, to increase yields, and to mitigate greenhouse-gas emissions and pollution of water and soil. Rhizobia and plant-growth-promoting rhizobacteria (PGPR) have been broadly used in agriculture, and advances in our understanding of plant-bacteria interactions have been achieved; however, the use of signaling molecules to enhance crop performance is still modest. In this study, we evaluated the effects of concentrated metabolites (CM) from two strains of rhizobia—*Bradyrhizobium diazoefficiens* USDA 110^T^ (BD1) and *Rhizobium tropici* CIAT 899^T^ (RT1)—at two concentrations of active compounds (10^–8^ and 10^–9^ M)—on the performances of two major plant-microbe interactions, of *Bradyrhizobium* spp.-soybean (*Glycine max* (L.) Merr.) and *Azospirillum brasilense*-maize (*Zea mays* L.). For soybean, one greenhouse and two field experiments were performed and effects of addition of CM from the homologous and heterologous strains, and of the flavonoid genistein were investigated. For maize, three field experiments were performed to examine the effects of CM from RT1. For soybean, compared to the treatment inoculated exclusively with *Bradyrhizobium*, benefits were achieved with the addition of CM-BD1; at 10^–9^ M, grain yield was increased by an average of 4.8%. For maize, the best result was obtained with the addition of CM-RT1, also at 10^–9^ M, increasing grain yield by an average of 11.4%. These benefits might be related to a combination of effects attributed to secondary compounds produced by the rhizobial strains, including exopolysaccharides (EPSs), plant hormones and lipo-chitooligosaccharides (LCOs). The results emphasize the biotechnological potential of using secondary metabolites of rhizobia together with inoculants containing both rhizobia and PGPR to improve the growth and yield of grain crops.

## Introduction

Sustainability probably represents the greatest challenge to increase food production. Year after year, the agricultural sector is forced to adopt new technologies to maintain high yields—without clearing new land for agriculture—and to minimize degradation of land that is occurring worldwide. Since the Green Revolution, the use of chemical fertilizers has played a key role in increasing yields; however, costs are often a major limitation to farmers in developing and poor countries, whereas, for developed countries, pollution of water and soil by fertilizers and greenhouse-gas emissions are sources of concern.

Bio-fertilizers can help meet the demands of sustainable, productive agriculture at low cost. Rhizobial inoculants have been applied to legume crops for over 120 years as bio-fertilizers, and inoculants carrying plant-growth-promoting rhizobacteria (PGPR) have been used in agriculture for over half a century (Okon and Labandera-Gonzalez 1994; Bashan and Bashan 2005; Hungria et al. 2005; Ormeño-Orrillo et al. 2012a). Considered safe, inoculants have been the focus of hundreds of basic and applied studies.

For rhizobial inoculants, a molecular dialogue between the host plant and the bacterium results in root nodulation and nitrogen fixation, involving plant flavonoids and bacterial nodulation (Nod) factors, identified as lipo-chitooligosaccharides (LCOs) (Schultze and Kondorosi 1996; Hungria and Stacey 1997; Perret et al. 2000; Oldroyd and Downie 2008; Ferguson et al. 2010); however, the roles of other molecules, such as those related to type-III secretion systems and exopolysaccharides (EPSs) (Perret et al. 2000; Fauvart and Michiels 2008; Downie 2010) have also been emphasized.

A broad range of beneficial effects has been reported for PGPR, including biological nitrogen fixation (Ashraf et al. 2011), phosphate solubilization (Rodriguez et al. 2004), and production of hormones, such as auxins, cytokinins, gibberelins and ethylene (Tien et al. 1979; Bottini et al. 1989; Strzelczyk et al. 1994) and control of pathogens (Araújo et al. 2005; Hernandez-Rodriguez et al. 2008; Wang et al. 2009), among others. However, our understanding of the molecular interactions of host plants with PGPR is still modest.

Despite results showing benefits of specific molecules to the performance both of rhizobia, e.g. by a supply of the flavonoids to soybean (*Glycine max* (L.) Merr.) and common bean (*Phaseolus vulgaris* L.) (Hungria and Phillips 1993; Hungria and Stacey 1997), and of PGPR, e.g. by a supply of crude or formulated metabolites of *Bacillus subtilis* (Araújo and Hungria 1999), the use of molecules to enhance crop performance under field conditions is incipient, highlighting the imbalance between basic knowledge and exploitation of biotechnological products in agriculture.

One exception is commercially available inoculants for soybean crops carrying Nod factors (Supanjani et al. 2005; Smith et al. 2012); however, responses in the field have often been slight and/or erratic, or dependent on specific conditions (Leibovitch et al. 2002). It could be that the problem lies in applying single molecules and that improved results may accrue with crude or formulated metabolites carrying several molecules (e.g., Araújo and Hungria 1999).

In this study, we evaluated the use of concentrated rhizobial metabolites on the performances of the two major grain crops that are frequently inoculated in South America, the *Bradyrhizobium* spp.-soybean and *Azospirillum brasilense*-maize (*Zea mays* L.) associations.

## Materials and methods

### Bacterial strains

For maize (*Zea mays* L.), liquid inoculants were prepared with *Azospirillum brasilense* strains Ab-V5 and Ab-V6, each at a concentration of 2 × 10^8^ cells mL^–1^. These strains, identified in a previous selection program (Hungria et al. 2010), are broadly used in commercial inoculants in Brazil.

For soybean [*Glycine max* (L.) Merr.], liquid inoculants were prepared with *Bradyrhizobium* strains CPAC 15 (=SEMIA 5079) and CPAC 7 (=SEMIA 5080), the combination most used in commercial inoculants in Brazil (Hungria et al. 2006), each at a concentration of 5 × 10^9^ cells mL^–1^. Recently, strains belonging to *Bradyrhizobium japonicum* have been split into two species, *B. japonicum* and *B. diazoefficiens*. CPAC 15 is still classified as *B. japonicum*, but CPAC 7 now belongs to *B. diazoefficiens*, and the type strain for this new species is USDA 110^T^ (Delamuta et al. 2013).

For the production of concentrated metabolites (CM), a search was performed among more than fifty strains of bacteria in the culture collections of the Universidad de Sevilla and of Embrapa Soja. Several properties potentially beneficial for plant growth were investigated, including the production of plant hormones (indole acetic acid, cytokinin, gibberelin), production of EPSs, and capacity to enhance soybean nodulation under controlled conditions. Two strains were identified: *Rhizobium tropici* CIAT 899^T^ and *B. diazoefficiens* USDA 110^T^, here named RT1 and BD1, respectively.

### Concentrated metabolites (CM)

CM were produced from RT1 and BD1, grown under conditions that enhance production of molecules beneficial to plant growth, as described before (Dardanelli et al. 2012). Metabolites were lyophilized, and effects of protectors, such as carbon methyl cellulose (CMC), bovine serum albumin (BSA) and milk powder were investigated. The process of producing the concentrated metabolites maintaining more than 90% of the original properties is now under registration. Shelf-life of the lyophilized CM was confirmed for 24 months, when the activity corresponded to 90% of that of the fresh metabolites.

### Seed inoculation

Prior to sowing, CM were re-suspended in a mixture of acetonitrile and water at concentrations of 0.1 mL L^-1^ and 1 mL L^-1^ of inoculant, corresponding to approximately 10^–9^ and 10^–8^ M, respectively. Inoculants for soybean (with *B. japonicum* and *B. diazoefficiens*) and for maize (with *A. brasilense*) were tested with and without CM. For soybean, homologous and heterologous CM were used, i.e., BD1 and RT1, respectively, while for maize only RT1 was evaluated.

Inoculants were applied to supply approximately 1.2 × 10^5^ cells of *A. brasilense* per seed of maize, and of 1.2 × 10^6^ cells of *Bradyrhizobium* spp. per seed of soybean, as recommended for both crops in Brazil.

For the greenhouse experiments, soybean seeds were surface-sterilized (Vincent 1970), and then inoculated by mixing the liquid inoculants with the seeds. For the field experiments, soybean and maize seeds were not surface sterilized and the inoculants were mixed with the seeds immediately before sowing. CM were added to the inoculants before seed inoculation.

### Greenhouse experiment

Differences in soybean nodulation may be difficult to detect in soils with indigenous or naturalized populations of compatible bradyrhizobia, as is the case for most soils in Brazil cropped with this legume. Therefore, for the soybean, one experiment was also performed under greenhouse conditions, using modified Leonard jars (Vincent 1970) containing sterilized substrate, consisting of a mixture of sand and pulverized coal (1:1, v/v) with application of N-free nutrient solution (Andrade and Hamakawa 1994).

The experiment consisted of seven treatments, including a non-inoculated control and all the others inoculated with *B. japonicum* and *B. diazoefficiens* strains CPAC 15 and CPAC 7, respectively, supplied or not with genistein (5 μM), or with CM-BD1 or CM-RT1, at 0.1 or 1.0 mL L^–1^. The jars were arranged in a completely randomized block design with six replicates. Four seeds of soybean cultivar BRS 245 were sown per jar and thinned to two plants five days after emergence. Mean temperatures during the experiments were of 28/23°C (day/night), and the N-free nutrient solution was applied as needed.

Plants were harvested at 45 days after emergence. Shoots were detached at the cotyledonary nodes, roots were washed, and nodules were removed and counted. Weight of shoots, roots and nodules were determined after drying to constant weight at 65°C (approximately 72 h). Shoots were ground (20 mesh) and total N was determined by Kjeldahl’s digestion method followed by the indophenol-blue colorimetric assay (Feije and Anger 1972).

### Field experiments

#### Site descriptions

Two field experiments were conducted with soybean and three with maize in the summer cropping season of 2011/2012. The soybean experiments were performed in Bonito, State of Mato Grosso do Sul (central-west region), and Ponta Grossa, State of Paraná (southern region). Maize experiments were performed in Bonito, Ponta Grossa, and Três Lagoas, State of Mato Grosso do Sul.

In Bonito (21º07' S and 56º28' W) the area is at an altitude of 290 m and the soil is classified as Latossolo Vermelho Distrófico (Brazilian classification) (Typic Haplustox, Soil Taxonomy, USDA). The climate is classified as *Aw* (Köppen’s classification) (tropical with dry winter). In Ponta Grossa (25°13' S and 50°1' W), at 880 m altitude, the climate is classified as *Cfb* (temperate with mild summer). The trials were performed on a Latossolo Vermelho Distrófico (Brazilian classification) (Typic Hapludox, Soil Taxonomy, USDA). In Três Lagoas, altitude 310 m, the climate is *Aw* and the soil is also a Latossolo Vermelho Distrófico (Typic Haplustox).

At each site, two months before the experiments were planted, twenty soil samples (0–20 cm depth) were taken to evaluate chemical properties (Pavan et al. 1992), as described before (Hungria et al. 2006). For chemical analyses, the samples were previously dried (60°C for 48 h) and sieved (2 mm). Soil texture was determined after Embrapa (1997).

Soil chemical properties and granulometric fractions at each site are shown in Table 1. About fifty days before starting the experiment, lime was applied to alleviate acidity, based on soil pH values. The amount of applied lime was estimated for a base saturation of 70% to increase the pH to approximately 5.5.

**Table 1:** Chemical and granulometric properties (0–20 cm) of the soils where the field experiments were performed

**Site**	**Chemical**										**Granulometry**		
	**pH**^**1**^	**P**	**H + Al**	**Al**	**K**	**Ca + Mg**	**SB**^**2**^	**CEC**^**2**^	**BS**^**2**^	**C**	**Clay**	**Silt**	**Sand**
	**CaCl**_**2**_	**mgdm**^**–3**^	**--------cmol**_**c**_**dm**^**–3 **^**---------**						**%**	**g dm**^**–3**^	**-------- g kg**^**–1 **^**--------**		
Bonito	4.22	1.94	8.42	1.02	0.45	3.98	4.43	12.85	34	7.7	162	266	572
Ponta Grossa	5.68	2.55	3.63	0.00	0.11	4.55	4.66	8.29	56	21.7	238	30	732
Três Lagoas	5.23	7.04	2.95	0.00	0.08	1.93	2.01	4.95	40	25.7	86	44	870

^1^Before addition of lime.

^2^SB, sum of bases; CEC, cation exchange capacity; BS, bases saturation = [(K + Ca + Mg)/Tcec] × 100, where Tcec = K + Ca + Mg + total acidity at pH 7.0 (H + Al).

In the experiments with soybean, the soil soybean-bradyrhizobial population was estimated using the most probable number (MPN) technique (Vincent 1970) and the statistical tables of Andrade and Hamakawa (1994) with soybean plants of cultivar BMX Potência RR® (BRASMAX) as trap host (Table 2).

**Table 2:** Agronomic information about the field trails

**Site**	**Crop**	**Soybean bradyrhizobia population (CFU g**^**–1 **^**soil)**	**Cultivar/Hybrid**	**Sowing**	**Harvest**	**Harvested area**
Bonito	Soybean	<10	BMX Potência RR	10/27/2011	03/20/2010	7.5 m^2^
	Maize	not evaluated	DKB 350 YG	10/28/2011	03/20/2012	8.4 m^2^
Ponta Grossa	Soybean	9.32 × 10^2^	BMX Potência RR	11/23/2011	04/03/2012	7.5 m^2^
	Maize	not evaluated	DKB 350 YG	11/24/2011	05/30/2012	8.4 m^2^
Três Lagoas	Maize	not evaluated	DKB 350 YG	11/03/2011	02/29/2012	8.4 m^2^

### Treatments and experimental design

For soybean, seven treatments were evaluated, consisting of a non-inoculated control and six treatments inoculated with *B. diazoefficiens* strain CPAC 7 + *B. japonicum* strain CPAC 15, supplied or not with genistein (5 μM), and CM of the homologous (CM-BD1) or heterologous (CM-RJ1) species, at the 0.1 and 1.0 mL L^–1^ of inoculant (10^–9^ and 10^–8^ M, respectively). The cultivar used was BMX Potência RR®.

The experiment with maize consisted of six treatments. Three treatments were non-inoculated and receiving 0, 75 and 100% of the dose of N-fertilizer at the V4 stage, as specified in the field management item. The other three treatments received 75% of the dose of N-fertilizer, were inoculated with *A. brasilense* strains Ab-V5 and Ab-V6 and received or not CM of *R. tropici* (CM-RT1) at 0.1 and 1.0 mL L^–1^ (10^–9^ and 10^–8^ M, respectively). The hybrid maize used was DKB 350 YG (DEKALB).

The experimental design was a completely randomized block with six replicates. For soybean, each plot had eight rows, spaced by 0.5 m, with 4 m (width) × 6 m (length) (24 m^2^). For maize, the plots had six rows, spaced 0.8 m, with 4.8 m (width) × 6 m (length) (28.8 m^2^). Plots were separated by at least 1.0 m and, where necessary, small terraces of approximately 1.5 m width were built to prevent contamination by superficial run-off containing bacteria or fertilizer, caused by heavy rains that often occur in the summer season.

### Field management

Densities were of about 300,000 plants ha^-1^ for soybean and of 60,000 plants ha^-1^ for maize. For both crops, 300 kg ha^–1^ of N-P-K (0-28-20) were applied in-furrow immediately before sowing. For soybean, no N-fertilizer was applied, and at V4 stage [four nodes on the main stem with fully developed leaves, beginning with the unifoliolated node (Fehr and Caviness 1977), approximately 30 days after emergence] plants received 20 g ha^–1^ of Mo (as Na_2_MoO_4_.2H_2_O) and 2 g ha^–1^ of Co (as CoCl_2_.6H_2_O) as foliar spray. For maize, 24 kg of N ha^–1^ (urea) were applied to all treatments at sowing, in-furrow, and, 30 days after emergence, plants received 0, 75 or 100% of the recommended dose for the crop, of 90 kg of N ha^–1^ (urea), broadcast.

For both crops, herbicides were used equally in all treatments, while insects were controlled with biological and chemical insecticides. Sowing and harvesting days and harvested area to evaluate grain yield in each experiment are shown in Table 2.

### Plant sampling, harvesting and analyses

For the soybean, at the V4 stage (Fehr and Caviness 1977) six plants were randomly collected per plot (avoiding central rows, to be used for determination of grain yield) for evaluation of nodulation [nodule number (NN) and nodule dry weight (NDW) per plant]. At R2 stage (full bloom), another six plants were collected for evaluation of shoot dry weight (SDW) and total N in shoot (TNS). Dry weight was determined as described for the greenhouse experiment. The early evaluation of nodulation at V4 indicates effects of inoculation, since nodules formed later result from infection also by the indigenous rhizobial population. Shoots were ground (20 mesh) and TNS determined after Kjeldahl’s digestion method, as described for the greenhouse experiment.

Maize plants were harvested at V4 stage (Iowa State University 1993) (fourth leaf fully expanded, approximately 35 days after emergence) for evaluation of SDW and TNS.

Grain yields of soybean and maize were determined at physiological maturity by harvesting a central area of each plot (Table 2). Grains were cleaned and weighed, with moisture content corrected to 13%.

### Statistical analyses

Data from each experiment were first submitted to tests of normality and homogeneity of variances for each variable and then to analysis of variance (ANOVA). When confirming a statistically significant value in the F test (*p* ≤ 0.05), a *post hoc* test (Duncan’s multiple-range test at *p* ≤ 0.05) was used as a multiple comparison procedure (SAS 1999).

## Results

Effects of inoculation with *Bradyrhizobium* spp. and of the supply of genistein or CM of rhizobia on soybean nodulation, growth and grain yield

Under greenhouse-controlled conditions and with a sterile substrate, soybean nodulation was significantly improved when, in addition to the inoculation with *B. diazoefficiens* + *B. japonicum*, seeds were supplied with genistein (5 μM), CM of the homologous strain (CM-BD1) (Table 3). The best performance was achieved with the addition of 1 mL L^–1^ CM-BD1, statistically increasing nodule number (NN) and dry weight (NDW) by 21% and 12%, respectively, in comparison to sole inoculation with *Bradyrhizobium*. CM-BD1 (1 mL L^–1^) also resulted in higher values, although without statistical difference, of shoot and root dry weight (SDW, RDW), N content and total N in shoots (NC, TNS). In contrast, the CM of the heterologous strains (CM-RT1) did not improve nodulation (Table 3).

**Table 3:** **Nodulation [nodule number (NN) and dry weight (NDW)], plant growth [shoot and root dry weight (SDW, RDW)], N concentration (NC) and total N accumulated in shoots (TNS) of soybean cultivar BRS 245 inoculated or not with *Bradyrhizobium japonicum *CPAC 15 +** ***B. diazoefficiens *CPAC 7 and supplemented or not with genistein (5 μM) or concentrated metabolites (CM) of *B. diazoefficiens *USDA 110 (BD1) or *Rhizobium tropici *CIAT 899 (RT1)**

**Treatment**	**NN**	**NDW**	**SDW**	**RDW**	**NC**	**TNS**
	**(# pl**^**–1**^**)**	**(mg pl**^**–1**^**)**	**(g pl**^**–1**^**)**	**(g pl**^**–1**^**)**	**(mg N g**^**–1**^**)**	**(mg N pl**^**–1**^**)**
Non-inoculated control	zero^2^	zero	0.81 b	0.51 b	6.57 b	5.19 b
Inoculated with *Bradyrhizobium*	38.0 b	162 a	1.31 a	0.77 a	19.38 a	25.5a
Inoculated + genistein	45.2 a	169 a	1.29 a	0.72 a	21.28 a	28.3 a
Inoculated + CM-BD1 (1 mL L^–1^)^1^	45.9 a	180 a	1.43 a	0.84 a	21.57 a	29.3 a
Inoculated + CM-BD1 (0.1 mL L^–1^)	42.5 a	140 a	1.22 b	0.63 ab	19.07 a	23.7 a
Inoculated + CM-RT1 (1 mL L^–1^)	33.2 b	138 a	1.23 b	0.66 ab	20.65 a	25.3 a
Inoculated + CM-RT1 (0.1 mL L^–1^)	32.5 b	128 a	1.14 a	0.61 ab	19.81 a	22.9 a

^1^ 0.1 and 1.0 mL L^–1^ correspond to approximately 10^–9^ and 10^–8^ M of active compounds, respectively.

^2^ Data represent the means of six replicates and when followed by different letters within the same column are significantly different (*p* ≤ 0.05, Duncan’s test).

Experiment performed in Leonard jars, with sterile substrate and receiving N-free nutrient solution, under greenhouse conditions. Plants were harvested at 45 days after emergence.

In the field trial carried out in Bonito, high NDW at the V4 stage and the highest grain yield were achieved with the addition of 0.1 mL L^–1^ CM-BD1 (Table 4). Grain yield increase in this treatment was significantly higher in comparison to both the non-inoculated control nodulated by naturalized bradyrhizobial strains (205 kg ha^–1^ or 7.6%), and the treatment inoculated only with *Bradyrhizobium* (203 kg ha^–1^ or 7.5%). At 1 mL L^–1^ of CM-BD1, the increase in grain yield in comparison to the treatment with sole inoculation with *B. japonicum* was of 47 kg ha^-1^ (1.7%), statistically non-significant*.* Interestingly, the highest SDW at R2 stage was achieved with CB-RT1, also at the lower dose, but a strong inhibitory effect was observed with the higher dose of 1 mL L^–1^ (Table 4).

**Table 4:** **Nodulation [nodule number (NN) and dry weight (NDW)], shoot dry weight (SDW), N concentration (NC) and total N accumulated in shoots (TNS) and grain yield of soybean cultivar BMX Potência RR inoculated or not with *Bradyrhizobium japonicum *CPAC 15 +** ***B. diazoefficiens *CPAC 7 and supplemented or not with genistein (5 μM) or concentrated metabolites (CM) of *B. diazoefficiens *USDA 110 (BD1) or *Rhizobium tropici *CIAT 899 (RT1). Experiments performed in two field sites in Brazil**

**Treatments**	**Bonito**						**Ponta Grossa**					
	**V4**		**R2**			**Maturity**	**V4**		**R2**			**Maturity**
	**NN**	**NDW**	**SDW**	**NC**	**TNS**	**Yield**	**NN**	**NDW**	**SDW**	**NC**	**TNS**	**Yield**
	**(# pl**^**–1**^**)**	**(mg pl**^**–1**^**)**	**(g pl**^**–1**^**)**	**(mg N g**^**–1**^**)**	**(mg N pl**^**–1**^**)**	**(kg ha**^**–1**^**)**	**(# pl**^**–1**^**)**	**(mg pl**^**–1**^**)**	**(g pl**^**–1**^**)**	**(mg N g**^**–1**^**)**	**(mg N pl**^**–1**^**)**	**(kg ha**^**–1**^**)**^**`**^
Non-inoculated control	10.4 ab^2^	43.2 b	18.4 ab	20.2 a	371 b	2701 b	45.7a	163 ab	10.4 ab	36.5 a	379 a	3166 a
Inoculated with *Bradyrhizobium*	15.1 ab	68.4 a	13.3 ab	20.4 a	271 c	2703 b	42.0 a	149 b	10.4 ab	35.1 ab	365 a	3191 a
Inoculated + genistein	10.7 ab	43.6 b	14.4 ab	20.2 a	290 c	2737 ab	48.7a	171 ab	12.3 a	32.0 b	393 a	3337 a
Inoculated + CM-BD1 (1 mL L^–1^)^1^	18.2 a	65.4 a	17.4 ab	17.6 a	306 c	2750 ab	52.5 a	206 a	12.1 a	34.6 ab	418 a	3359 a
Inoculated + CM-BD1 (0.1 mL L^–1^)	16.0 a	68.1 a	19.8 ab	17.6 a	348 b	2906 a	57.8 a	212 a	11.3 ab	34.6 ab	390 a	3275 a
Inoculated + CM-RT1 (1 mL L^–1^)	7.3 b	25.1 c	10.4 b	16.8 a	174 d	2641 b	45.3 a	170 ab	9.2 ab	33.4 ab	307 a	3202 a
Inoculated + CM-RT1 (0.1 mL L^–1^)	13.4 ab	69.9 a	23.4 a	18.1 a	423 a	2817 ab	46.8 a	162 ab	8.9 b	35.3 ab	314 a	3253 a

^1^ 0.1 and 1.0 mL L^–1^ correspond to approximately 10^–9^ and 10^–8^ M of active compounds, respectively.

^2^ Data represent the means of six replicates and when followed by different letters within the same column are significantly different (*p* ≤ 0.05, Duncan’s test).

In Ponta Grossa, highest NDW at V4 was obtained with both doses of CM-BD1, but with no statistical difference in comparison to the naturalized population and to the control inoculated solely with *Bradyrhizobium* (Table 4). Additionally, although without statistical difference, an increase of 169 kg ha^-1^ (5.2%) was observed in grain yield in the treatment receiving 1 mL L^–1^ of CM-BD1, and of 84 kg ha^-1^ (2.6%) at the lower dose of 0.1 mL L^–1^. In this experiment, the addition of genistein also improved yield of inoculated plants by 146 kg ha^-1^ (4.5%), but the effect was not statistically significant. A decrease in SDW at R2 stage was observed with the supply of heterologous CM-RT1, but no differences in total N accumulation in shoots was observed (Table 4).

When the experiments were analyzed together, considering the treatment inoculated with *Bradyrhizobium* as the control, the addition of the lower dose of active compounds (0.1 mL L^–1^) resulted in increases of 29.0% in NN (*p* ≤ 0.05, Duncan’s test) and of 143.5 kg ha^-1^ (4.8%) in grain yield (*p* ≤ 0.09, Duncan’s test).

Effects of inoculation with *A. brasilense* and of CM of rhizobia on maize growth and grain yield

Under field conditions, in Bonito, at V4 stage, no differences were observed in SDW, but the highest N content (TNS) (although not differing statistically) was achieved with the CM-RT1 at the lower concentration (0.1 mL L^-1^) (Table 5). At physiological maturity, the comparison of the treatments receiving 75% of N revealed that plants inoculated with *A. brasilense* and supplied with 0.1 mL L^–1^ of CM-RT1 resulted in a significant increase in grain yield (1,045 kg ha^-1^, or 19%) and, although not statistically significant, of 936 kg ha^-1^(17%) in relation to the inoculated treatment without CM-RT1 (Table 5); for this last comparison, differences were significant at *p* ≤ 0.07 (Duncan’s test).

**Table 5:** **Shoot dry weight (SDW) and total N accumulated in shoots (TNS) at V4 and grain yield at the maturity of maize hybrid DKB 350 YG inoculated or not *Azospirillum brasilense *strains Ab-V5 + Ab-V6 and supplemented or not with concentrated metabolites of *Rhizobium tropici *CIAT 899 (CM-RT1)**

**Treatment/Site**	**Bonito**			**Ponta Grossa**			**Três Lagoas**		
	**V4**			**V4**			**V4**		
	**SDW**	**TNS**	**Yield**	**SDW**	**TNS**	**Yield**	**SDW**	**TNS**	**Yield**
	**(g pl**^**–1**^**)**	**(mg pl**^**–1**^**)**	**(kg ha**^**–1**^**)**	**(g pl**^**–1**^**)**	**(mg pl**^**–1**^**)**	**(kg ha**^**–1**^**)**	**(g pl**^**–1**^**)**	**(mg pl**^**–1**^**)**	**(kg ha**^**–1**^**)**
0%N	13.1 a	402 a	5452 b	17.2 c	255 c	5708 c	7.1 c	161 b	2444 b
100%N	16.1 a	510 a	6184 ab	26.4 ab	558 ab	8483 a	9.7 a	252 a	2965 ab
75% N	12.4 a	407 a	5516 b	24.2 ab	511 b	7964 ab	9.4 ab	243 a	3010 ab
75% N + Inoculated with *A. brasilense*	14.0 a	412 a	5625 ab	22.1 bc	445 b	7208 b	7.9 bc	201 b	3319 a
75% N + Inoculated with *A. brasilense* + CM-RT1 (1 mL L^–1^)	11.5a	399 a	6091 ab	25.5 ab	542 ab	7707 ab	7.0 c	172 b	2972 ab
75% N + Inoculated with *A. brasilense* + CM-RT1 (0.1 mL L^–1^)	12.9 a	458 a	6561 a	29.5 a	669 a	8113 a	7.9 bc	199 b	3322 a

^1^ 0.1 and 1.0 mL L^–1^ correspond to approximately 10^–9^ and 10^–8^ M of active compounds, respectively.

^2^ Data represent the means of six replicates and when followed by different letters within the same column are significantly different (*p* ≤ 0.05, Duncan’s test).

All plants received 24 kg of N ha^-1^ at sowing and 0, 75 or 100% of N (90 kg of N ha^-1^) at 30 days after emergence, broadcasted. Experiment performed in three field sites in Brazil.

In Ponta Grossa, the best performance was achieved again in the treatment inoculated with *A. brasilense* supplied with CM-RT1 (0.1 mL L^–1^), resulting in higher TNS at V4, statistically similar to the non-inoculated control receiving 100% of N-fertilizer (Table 5). In the comparison of the treatments receiving 75% of N-fertilizer, the highest grain yield was also observed in the treatment inoculated with *A. brasilense* supplied with 0.1 mL L^–1^ of CM-RT1 (Table 5).

A severe drought in Três Lagoas inhibited plant growth and reduced grain yield. Under these conditions, plant biomass at V4 was improved by addition of the full dose of N-fertilizer (Table 5). However, at the final harvest, higher grain yields were observed in the plants inoculated with *A. brasilense*, with and without CM. Although not differing statistically from the non-inoculated control with 75% of N-fertilizer, these two treatments increased grain yield by an average of 300 kg ha^–1^ (Table 5).

Considering the overall analysis of the three field experiments, statistically significant increases were obtained with the supply of 0.1 mL L^–1^ CM-RT1, of 614 kg ha^-1^ (11.4%), when compared to the treatment inoculated solely with *A. brasilense* and receiving 75% of N-fertilizer (*p* ≤ 0.05, Duncan’s test).

## Discussion

Modern agriculture has increasingly focused on the use of microbial products as alternatives to chemical fertilizers. Benefits from this replacement include substantially lower costs for farmers, less pollution and land degradation, and reduced concerns regarding adverse side effects on human health (Crews and Peoples 2004; Peoples et al. 2009; Saharan and Nehra 2011; Bakker et al. 2012). It is noteworthy that extensive use of rhizobial inoculants in Brazil, mainly with soybean, provides N with a value equivalent to US$ 7 billion in fertilizers (Hungria et al. 2006; Hungria et al. 2007). Several countries—including Brazil—benefit from the use of inoculants carrying PGPR, which may benefit crops by promoting uptake of nutrients and by increasing resistance to abiotic and biotic stresses, among other effects (Okon and Labandera-Gonzalez 1994; Bacon and Hinton 2002; Bashan et al. 2004; Bashan and Bashan 2005; Hungria et al. 2010; Saharan and Nehra 2011). Currently, in Brazil, about 25 million doses of rhizobial inoculant for soybean and 2 million doses of inoculant containing *A. brasilense* for maize and wheat crops are produced annually. However, despite improved understanding, particularly over the past two decades, of molecular signaling in the rhizobia-legume interaction (e.g. Geurst and Bisseling 2002; Brencic and Winans 2005; Ferguson et al. 2010), as well as of other signals involved in host-microbe interactions (Brencic and Winans 2005), transfer of this knowledge to effective commercial products is still incipient.

In our study, we investigated the effects of supplying inoculants carrying *Bradyrhizobium* spp. and *A. brasilense* strains with concentrated metabolites (CM) of selected rhizobial strains. For soybean, under greenhouse and sterile-substrate conditions we found that addition of the homologous concentrated metabolites (CM-BD1) at the higher dose (1 mL L^–1^ of inoculant) increased nodule number in comparison to the treatment inoculated solely with *Bradyrhizobium*. Considering the field experiments individually, both doses of CM-BD1 (0.1 and 1.0 mL L^–1^, corresponding to 10^–9^ and 10^–8^ M of active compounds, respectively) resulted in improvements in plant growth. Considering both field experiments, the addition of the lower dose resulted in increases of 23.6% in NN, and of 4.8% in grain yield. Interesting, positive effects were observed only with the metabolites from the homologous species, and the higher dose of the heterologous RT1 negatively affected nodulation and plant growth.

Secondary metabolites may provide an evolutionary advantage for survival of microbes in soil (Demain 1998), and they may also help in the establishment of symbiotic partnerships (Brencic and Winans 2005). Chemical analyses of the secondary metabolites contained in CM-BD1 and CM-RT1 indicated that the benefits may result mainly from LCOs, but also from EPSs and plant hormones.

EPSs play important roles at several stages of the development of the root-nodule symbiosis (Fraysse et al. 2003; Kirichenko et al. 2004; Becker et al. 2005; Downie 2010). Therefore, the addition of extra EPSs to inoculants may increase root infection. Furthermore, EPSs protect bacteria against stressful conditions, such as desiccation and osmotic and pH extremes, substantially increasing cell survival (Castellane and Lemos 2007); this feature may be critical for maintenance of bacterial viability in inoculants while on the shelf and after application to seeds or to the soil. Many plant-associated bacteria, including rhizobia, synthesize plant growth hormones, such as auxins (Tien et al. 1979; Ashraf et al. 2011), gibberelins (Bottini et al. 1989), cytokinins (Tien et al. 1979; Strzelczyk et al. 1994) and ethylene (Strzelczyk et al. 1994). Recently, genomic sequences of *R. tropici* strain CIAT 899^T^ and *Rhizobium* sp. PRF 81 highlighted a variety of metabolic pathways related to plant-hormone synthesis (Ormeño-Orrillo et al. 2012b). Therefore, EPSs and plant hormones may have contributed to the observed increases in soybean performance and yield.

Lipo-chitooligosaccharides affect a number of physiological processes in the legume host plant, including root-hair curling and stimulation of division of cortical cells (Schultze and Kondorosi 1996; Hungria and Stacey 1997; Perret et al. 2000; Oldroyd and Downie 2008; Ferguson et al. 2010). It has also been demonstrated that LCOs effects resemble those of cytokinins, and of the inhibitors of the transport of auxins (Relic et al. 1993); interestingly, they also activate enzymes related to plant defense (Inui et al. 1997). Finally, other benefits attributed to LCOs require further exploration, e.g., increases in seed germination (Miransari and Smith 2009). We attribute the effects observed in our study to LCOs; they are consistent with the activity of these compounds at concentrations as low as 10^–12^ M (Hungria and Stacey 1997). Activity at such low concentrations may be responsible for the differences observed between greenhouse and field experiments. In addition, it is known that the LCOs of *B. japonicum* are very specific, with a methyl-fucose moiety under the control of *nodZ* (López-Lara et al. 1996), which may explain the responses exclusively to homologous metabolites. Finally, in our study the addition of a plant molecular signal (genistein) was not as successful as the addition of LCOs, although the concentration applied (5 μM) was much lower than doses previously reported for soybean and common bean (40 μM) (Hungria and Stacey 1997). The effects of adding CM to the maize crop surpassed those observed with soybean. Considering three field experiments, increases in grain yield by the addition of CM-RT1 at the lower dose (10^–9^ M) were of 11.4%. The increases may be attributable to EPSs and plant hormones, and in the latter case, it is known that the effects are strongly dependent on concentration, and can be inhibitory at higher concentrations (Arshad and Frankenberger 1991), which may explain the better performance at the lower concentration of CM. In addition, benefits can also be attributed to the LCOs, which may activate cell division in non-leguminous plants, such as tobacco (*Nicotiana* sp.) (Baier et al. 1999), tomato (*Solanum lycopersicum*) (Staehelin et al. 1994), carrot (*Dacus carota*) (De Jong et al. 1993), and also maize (Khana et al. 2008). Often, the effects mimic those of plant hormones, such as cytokinins and auxins (Dyachok et al. 2000). Interestingly, under greenhouse conditions Souleimanov et al. (2002) reported increases in soybean and maize biomass seven days after the addition of LCO (at 10^–7^ M) of *B. japonicum*.

In conclusion, the results from our study indicate biotechnological potential in the use of secondary metabolites of rhizobia—together with inoculants containing both rhizobia and PGPR—to improve growth and grain yields of crops of soybean and maize. Such improvements, which favor agricultural sustainability by bringing economic and environmental benefits, merit further investigation. It is noteworthy that commercial products containing the CM of our study are now registered in Brazil.

## Competing interests

The authors declare that they have no competing interests.
